# Case of Diabetes, Cured by Bleeding, the Vapour Bath, &c.

**Published:** 1826-05

**Authors:** George Lefevre

**Affiliations:** Member of the Royal College of Physicians, London.


ART. III.—*Case of Diabetes, cured by Bleeding, the Vapour Bath, &c.* By GEORGE LEFEVRE, M.D. F.L.S. Member of the Royal College of Physicians, London. (Communicated by Dr. JAMES JOHNSON.)

LOUISA SUBIRON, aged fifty, of a melancholic temperament, and subject to frequent nervous attacks, but otherwise of a good constitution, and rather inclined to corpulence, became the subject of the following disease in the autumn of the year 1823.

She first applied to me in the month of June, and gave me this account of her symptoms, confessing at the same time that she had a long while concealed them, and that the distress she suffered now compelled her to seek medical assistance. She stated that for some time she had been tormented by excessive thirst, which she had endeavoured to allay, but in vain, by drinking copiously of refreshing beverages. The quantity of fluid which she drank in the course of the day, averaged from twelve to fourteen pints. She slept very badly, being obliged to drink frequently during the night; and, after some hours' sleep, her tongue was so parched that she could not articulate clearly till she had moistened her mouth. Her skin was dry and rough, without the least perspiration, which she could not excite by any means in her power. The bowels were obstinately constipated; the digestive functions much deranged, and the appetite very much impaired. The nervous system was much shaken. She complained of giddiness in the head and of dimness of sight: when she read, she said "she continually lost her place." She complained also of much trembling of the knees, and wept frequently. She made no mention of the quantity of urine she secreted, which, however, was very considerably greater than the fluid she drank. She concealed this, either from *mauvaise honte*, or, considering the thirst as the only disease, she paid little attention to this circumstance. As to her previous state of health, it appeared that she had been subject only to nervous affections, whose origin was attributable to mental causes. During the previous winter, however, she had suffered much from pains in her loins, which were relieved by warm baths and opiates. The cessation of the menses, which took place more than a year previous to her present attack, was not accompanied by any unpleasant symptom, though she herself dreaded this period, from the fear of becoming dropsical, as several of her relations had died of dropsy soon after that epoch.

Upon the first view of the case, I was inclined to consider the whole as an hysterical affection, and I prescribed her some warm baths, and gave her the nitric-acid drink, with opiates at night. This practice was continued two or three days, with some relief to the thirst, but she complained of her nerves being more agitated, and ascribed it to the use of the acid. I determined, therefore, to compare the quantity of fluid she drank with the urine. The latter was about one-sixth part more than the former. M. Chevreuil, a chemist of celebrity, analysed, and found that it contained a very large proportion of sugar. No doubt remained, therefore, as to the disease in question. The quantity of urine voided at this period amounted to fifteen pints per day; it was of a pale straw colour, sweetish to the taste, and had a nauseous sickly smell.

Notwithstanding the shattered state of the nervous system, I was resolved to bleed her; the more particularly as the disease had been preceded by pains in the loins. Twelve ounces of blood were taken from the arm, June 16, 1824. It coagulated slowly; the crassamentum was loose, and the serum like whey. She felt herself immediately relieved, slept better than she had done previously, and felt stronger and lighter than before. The thirst was considerably diminished; the tongue became moister, but the skin continued dry. She was requested to live upon animal food, with but a small quantity of bread. The warm bath was prescribed every evening. Ten grains of the compound powder of ipecacuanha were given at bed-time. The bowels were moved by means of colocynth and calomel; and she was desired to drink equal parts of lime-water and milk for her common drink. The quantity of urine voided the day previous to the bleeding amounted to twelve pints.

For some days all the symptoms rapidly diminished, but again returning, though in a diminished degree, the blood-letting was again repeated, and twelve ounces more were taken from the arm. It coagulated more rapidly than on the former occasion, and the crassamentum was much firmer, the serum more clear. (June 21.) She felt instantaneous relief, as before, expressing astonishment at feeling stronger after being bled.

Two days after the second bleeding, the urine was diminished more than a third; it was of a much deeper colour, and had more of an ammoniacal smell; it still yielded sugar on evaporation.

Not being able to excite perspiration, either by the use of the warm bath or by sudorifics given internally, and grounding the hopes of a cure principally upon the re-establishment of this function, I proposed to employ the vapour bath, and to endeavour to keep up the perspiration it might excite by hand-exercise. She was exposed (June 30,) during half an hour to vapour heated to 125° Fahrenheit. She perspired most profusely, and changed her linen and sheets three times during the evening. The following day she felt much more comfortable, but weak; she walked, however, a mile, warmly clad, and perspired freely. The urine was now reduced to five pints per day.

No change took place for several days; but, being prevented from taking exercise, on account of the rainy weather, her skin became again dry, and the vapour bath was repeated, July 10. She could not remain in the bath so long as before, on account of the profuse perspiration it occasioned. She felt weak for some days afterwards, and perspired freely without any bodily exertion.

All the symptoms of her disease seemed now to leave her: the quantity of urine was not greater than natural, and of a good colour; it yielded still a small quantity of sugar on evaporation. The digestive functions were quite restored. She slept better than she had done for years, and her spirits were wonderfully improved. She had gained also much strength, and walked two or three miles without being fatigued. She was now sent into the country, where she remained three months, improving generally in health.

She was attacked, in the month of August, with severe pains in her loins, such as she had before frequently experienced; the pains extended along the thighs as far as the knee. These symptoms were soon relieved by a blister applied to the loins, and by the use of the warm bath. Two days after, a diarrhœa came on, which soon, however, ceased. Since this period

(August 10, 1824,) she has complained of no unpleasant symptom. Every function performs its office naturally. She has taken no other medicine than a few opening pills during the last three months. At no period of the disease was the appetite the least increased.

As much change seems to have been produced upon her mind as her body : she is no longer gloomy and desponding, as heretofore, but gay and lively, and follows her usual occupation with more satisfaction than she had ever done.

The urine has been analysed to-day, (Nov. 8, 1824 :) it contains not a particle of sugar. An incurable disease completely

